# Post-booster longitudinal plasma proteomic changes following BNT162b2 COVID-19 vaccination in Qatar

**DOI:** 10.3389/fimmu.2026.1762522

**Published:** 2026-04-24

**Authors:** Sana Bentebbal, Ahmed Zaqout, Bakhita Meqbel, Ilham Bensmail, Abdullah Aldushain, Alberto de la Fuente, Remy Thomas, Adviti Naik, Hibah Shaath, Neyla S. Al-Akl, Abdi Adam, Houda Y. A. Moussa, Kyung C. Shin, Rowaida Z. Taha, Mohammed Abukhattab, Muna A. Al-Maslamani, Nehad M. Alajez, Abdelilah Arredouani, Yongsoo Park, Sara A. Abdulla, Omar M. A. El-Agnaf, Houari B. Abdesselem, Ali S. Omrani, Julie Decock

**Affiliations:** 1 Translational Oncology Research Center, Qatar Biomedical Research Institute (QBRI), Hamad Bin Khalifa University (HBKU), Qatar Foundation (QF), Doha, Qatar; 2 Communicable Disease Center, Hamad Medical Corporation (HMC), Doha, Qatar; 3 Proteomics Core Facility, Hamad Bin Khalifa University (HBKU), Qatar Foundation, Doha, Qatar; 4 Qatar Biomedical Research Institute (QBRI), Hamad Bin Khalifa University (HBKU), Qatar Foundation (QF), Doha, Qatar; 5 Diabetes Research Center, Qatar Biomedical Research Institute (QBRI), Hamad Bin Khalifa University (HBKU), Qatar Foundation (QF), Doha, Qatar; 6 Neurological Disorders Research Center, Qatar Biomedical Research Institute (QBRI), Hamad Bin Khalifa University (HBKU), Qatar Foundation (QF), Doha, Qatar; 7 College of Medicine, Qatar University, Doha, Qatar; 8 College of Health and Life Sciences (CHLS), Hamad Bin Khalifa University (HBKU), Qatar Foundation (QF), Doha, Qatar

**Keywords:** BNT162b2, booster, MMP-7, PARP-1, proteomic profiling, SARS-CoV-2

## Abstract

**Background:**

The COVID-19 pandemic imposed a major global health and economic burden. Although the pandemic was no longer declared a public health emergency of international concern in May 2023, SARS-CoV-2 variants continue to emerge, and millions remain affected by long COVID. This raises the question whether continued vaccination provides lasting benefits in preventing viral transmission and severe illness.

**Aim:**

This longitudinal study assessed the effects of the third BNT162b2 mRNA vaccine dose on the circulating proteome for 6 months.

**Methods:**

Plasma levels of 354 unique proteins were quantified before, and at 3- and 6-months post-booster using Olink technology in 70 healthy individuals; 35 infection-naïve and 35 previously infected individuals (18 infected before, 17 after completing the two-dose regimen).

**Results:**

Infection-naïve individuals showed altered levels of eleven and eight proteins at 3- and 6-months post-booster, respectively, including a significant sustained increase in PARP-1 (FC = 1.53, p=8.59x10^-5^, pFDR=0.01) and significant decrease in MMP-7 (FC = 0.68, p=4.58x10^-5^, pFDR=0.01), in addition to elevated levels of MMP-1 (FC = 1.46, p=0.04, pFDR>0.05) and decrease in 4E-BP1 (FC = 0.58, p=0.01, pFDR>0.05) at 6 months post-booster. Similarly, previously infected individuals, in particular those with earlier infections before receiving the second dose exhibited a significant sustained upregulation of PARP-1 (FC = 2.10, p=1.19x10^-5^, pFDR=0.003) and downregulation of MMP-7 (FC = 0.58, p=2.19x10^-5^, pFDR=0.003) at 6-months post-booster. Notably, PARP-1 and MMP-7 were consistently affected across all individuals. Longitudinal proteome profiling revealed dysregulation of key inflammatory proteins for up to 6 months post-booster, including PARP-1 and MMP-7 (pFDR=1.58x10^–8^ and pFDR=1.59x10^-5^, respectively).

**Conclusions:**

These findings provide insights into the temporal dynamics of circulating proteomic responses following booster vaccination, highlighting molecular features that may be relevant to immune readiness and post-vaccination inflammatory processes.

## Introduction

In December 2019, the emergence of severe acute respiratory syndrome coronavirus 2 (SARS-CoV-2) caused a highly transmissible respiratory illness, known as Coronavirus disease 2019 (COVID-19), which was subsequently declared a global pandemic in early 2020. Several vaccines, such as those by Pfizer-BioNTech (BNT162b2), Moderna (mRNA-1273), and AstraZeneca (ChAdOx1 nCoV-19) proved highly effective at preventing severe disease, hospitalization, and death (1–3). Although we have entered the post-COVID19 pandemic phase, the continuous emergence of new variants poses an ongoing challenge for vaccine efficacy, urging the need for development of novel vaccines that protect against newly circulating variants (4, 5). According to the latest recommendation of the WHO Technical Advisory Group on COVID-19 Vaccine Composition (TAG-CO-VAC), currently approved COVID-19 vaccines continue to provide effective immune protection against circulating variants of interest, further highlighting the importance of continued vaccination, particularly among individuals at high risk of severe COVID-19 (6). Furthermore, the natural waning of immunity underscores the importance of booster vaccination to maintain population-level protection (7–9). However, the economic burden of booster vaccination campaigns, together with their implications for healthcare systems such as long COVID management, warrants careful consideration. To date, it remains unclear whether proteomic and immunological markers can aid to stratify individuals that are more likely to benefit from booster vaccination strategies, thus optimizing resource allocation while maintaining effective population-level protection.

Blood proteomic profiling has emerged as a powerful tool to evaluate the molecular mechanisms of immune responses and inflammatory signaling following SARS-CoV-2 infection and vaccination. Previous studies have identified circulating protein signatures associated with COVID-19 disease severity and long-term complications, including neurological outcomes. Using the Olink proteomic platform, COVID-19 positive patients exhibited changes in the circulating protein levels of 269 out of 368 analytes compared to healthy control subjects (10). Of the 269 analytes, six immune-related proteins (IL6, CKAP4, Gal-9, IL-1ra, LILRB4 and PD-L1) were associated with disease severity. Individuals who survived COVID-19 have been found to display dysregulation of multiple inflammation-related molecules, reflecting systemic inflammation (11). Through blood proteome profiling, we previously identified a 12-protein signature that accurately stratified COVID-19 patients based on disease severity as well as a 34-neurological protein signature that correlated with various neurological diseases and could potentially be used to identify long-term COVID-19 associated neurological complications (12). Furthermore, distinct changes in proteins and metabolites have been observed following COVID-19 vaccination (13–21). For example, using an 7,289 aptamer-based proteomic assay, UBE2D1/UBB, CHAC1, and CTAG1A/CTAG1B were found to be elevated in sera from BNT162b2 vaccine recipients at 1-month post-third dose vaccination, concomitant with an upregulation of TICAM1 and RIP1 mediated IKK signaling (22). Furthermore, profiling of 67 cytokines in BNT162b2 vaccine recipients demonstrated an increase in IFN-γ and CXCL10 levels in addition to a higher frequency of CD14+CD16+ monocytes and innate immune response transcriptional signature following the second dose (13). Longitudinal analysis of high and low responders to the BNT162b2 vaccine revealed changes in both innate and adaptive immune responses with increased expression of interferon-driven genes and genes related to enhanced antigen presentation, in conjunction with robust cytokine responses related to Th1 differentiation (23). In addition, alterations in complement and coagulation pathways were observed in 6-months post-immunization serum samples of BNT162b2 vaccinated individuals with prior SARS-CoV-2 infection using a quantitative LC-MS/MS proteomics approach (15). Notably, multiplex LC-MS/MS identified that serum levels of the complement factor C1q are more strongly correlated with levels of neutralizing antibodies compared to IgG1 levels in response to infection with different variants of concerns (24). LC-MS/MS was also used to profile the plasma and urine proteomes of healthy individuals before and after receiving the first dose, and after the second dose of the BBIBP-CorV vaccine (25). This comprehensive analysis showed that plasma levels of ACE2-RBD-inhibiting antibodies associated with plasma protein changes related to activation of the LXR/FXR pathway and with urine proteome alterations related to the complement system, acute phase response signaling, and LXR/FXR and STAT3 pathways. Vaccination with the less widely used SARS-CoV-2 CoronaVac and Vero Cell vaccines was associated with changes in TCA cycle intermediates and amino acid metabolites, and routine clinical laboratory measurements such as hemoglobin A1c, electrolyte levels, coagulation profiles, and renal function-related proteins. Tandem Mass Tag (TMT)-based proteomic analysis of 1,715 serum proteins and 4,342 peripheral blood mononuclear cell (PBMC) proteins revealed that measurement of a set of 4 PBMC biomarkers (PYCARD, MTMR2, PPCDC, and BRAF) prior to vaccination with the Sinovac vaccine could predict seropositivity until 152 days after receiving the second vaccine dose (26). In addition, a set of seven serum proteins (SERPINA10, SOD3, LTA4H, SPP2, NAGLU, APLP2, and CHRDL2) accurately predicted antibody persistence until 28 days after the second dose.

**Figure 1:**
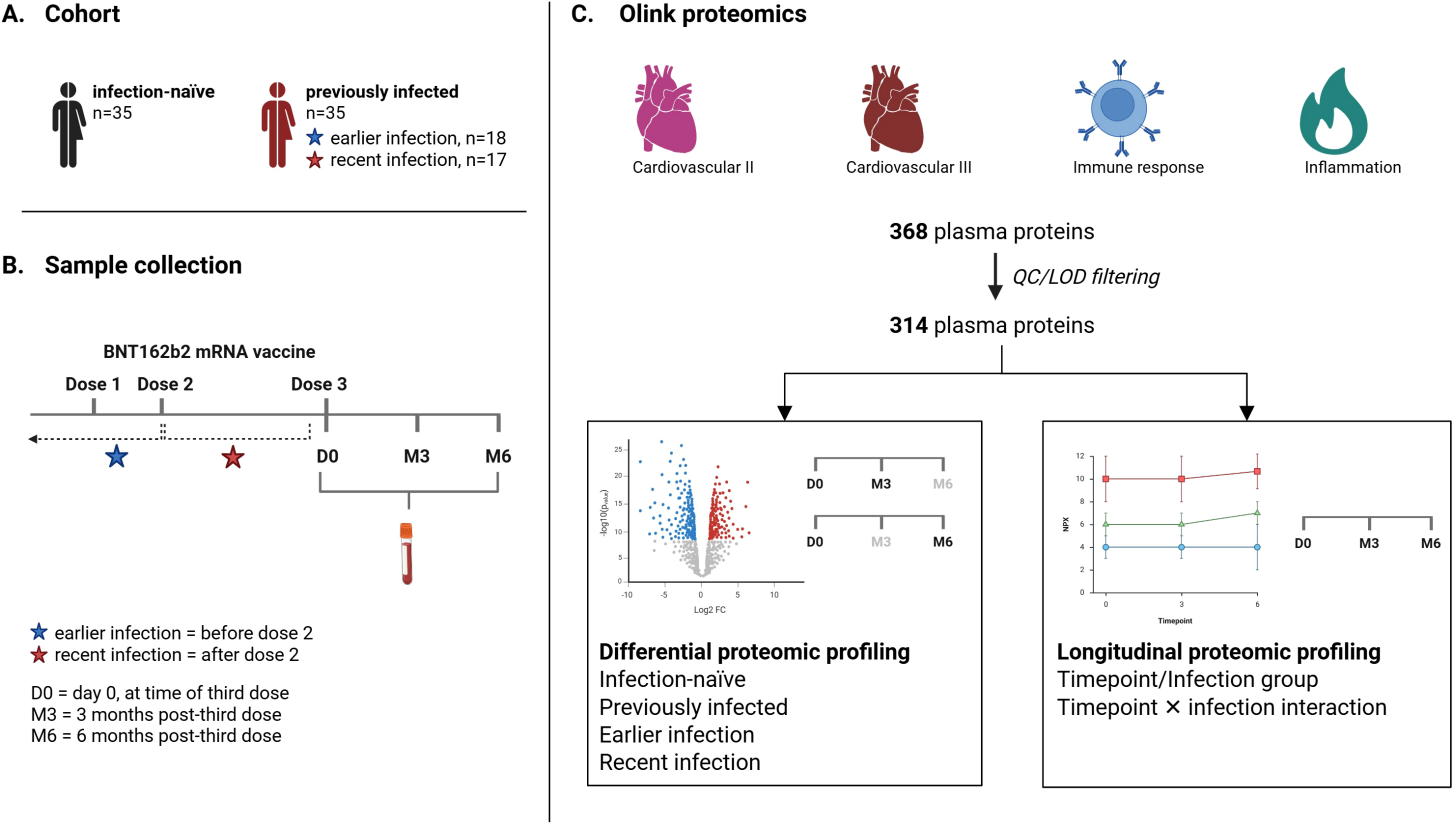
Schematic overview of the study design. **(A)** The study cohort included 70 healthy adults from the craft and worker community in Qatar who completed the two-dose BNT162b2 vaccination regimen. **(B)** Blood samples were collected at the time of administration of the third dose (booster, D0), and three (M3) and six months (M6) following the third dose. **(C)** Plasma levels of 368 proteins were measured using Olink proximity extension assay technology. Following preprocessing quality control steps including sample outlier identification and limit of detection-based filtering, LIMMA was used to analyze differential expression of 314 proteins between infection-naïve and previously infected individuals, stratified by time of infection. LME modeling was performed to assess longitudinal protein expression patterns, with timepoint, infection group and timepoint × infection status interaction set as fixed effects. LIMMA, Linear Models for MicroArray data; LME, Linear Mixed-Effects model; LOD, Limit of Detection; QC, Quality Control.

In this study, we build on our previous work examining longitudinal cellular and humoral immune responses following BNT162b2 booster vaccination in craft and manual workers from Qatar by providing a comprehensive analysis of circulating proteome changes over a six-month period following administration of the third vaccine dose (27). Previously, we demonstrated that BNT162b2 booster vaccination enhanced both humoral and cellular anti-viral immune responses, including sustained neutralizing antibody responses and increased T cell activity up to six months post-booster. Here, using the high-throughput Olink technology, we determined the expression of 314 proteins in the circulation of 70 individuals from the same cohort before, and three and six months after booster vaccination (**Figure 1**), assessing how pre-booster natural infection influences post-vaccination antiviral immune signaling and inflammatory processes. This longitudinal approach allowed us to identify persistent molecular signatures that reflect both vaccine-induced immunity and potential post-vaccination inflammatory effects, enhancing our understanding of immune readiness and systemic responses to SARS-CoV-2 vaccination.

## Materials and methods

### Study population

A total of 70 healthy adults from the craft and manual worker community in Qatar were included in the study (**Table 1**). As previously described, all individuals received two doses of the BNT162b2 mRNA vaccine and received the third dose at the Communicable Disease Center in Qatar as per the national vaccination program, at which time they were enrolled in the study (27). Suspected SARS-CoV-2 infections were tested by PCR and automatically updated in the electronic medical records as per the Qatar national testing framework. Demographic data, including information on PCR-confirmed SARS-CoV-2 infection were obtained from national centralized electronic medical records as previously described (27). Exclusion criteria included a confirmed SARS-CoV-2 infection within four weeks before receiving the third vaccine dose, immunocompromised status due to underlying disease or medical treatment, and pregnancy. None of the participants exhibited clinical symptoms at time of blood sampling. Individuals with a confirmed SARS-CoV-2 infection before receiving the second vaccine dose were classified as individuals with earlier infections, while those with infections after the second dose (and before the third dose) were grouped as individuals with recent infections.

**Table 1:** Study population demographics (27).

Demographic	All participants n (%)	Infection-naïve n (%)	Earlier infection n (%)	Recent infection n (%)
Prior SARS-CoV-2 infection *				
infection-naïve	35 (50)			
earlier infection	18 (26)			
recent infection	17 (24)			
Age				
18-49	64 (91)	33 (94)	14 (78)	17 (100)
50+	6 (9)	2 (6)	4 (22)	0
Sex				
female	6 (9)	0	3 (17)	3 (18)
male	64 (91)	35 (100)	15 (83)	14 (82)
Smoking history				
never smoked	41 (59)	19 (54)	12 (66)	10 (59)
former smoker	10 (14)	5 (14)	3 (17)	2 (12)
current smoker	19 (27)	11 (32)	3 (17)	5 (29)
Medical history				
diabetes mellitus	1 (1)	0	1 (6)	0
epilepsy	1 (1)	0	1 (6)	0
high blood pressure	2 (3)	0	2 (10)	0
hypothyroidism	2 (3)	0	1 (6)	0
none	64 (92)	35 (100)	13 (72)	17 (100)

*earlier infection refers to infections before receiving the second dose, while recent infections refer to infections after completion of the two-dose regimen.

### Sample collection and processing

Peripheral blood samples were collected in 10 ml EDTA blood tubes at three timepoints: at the time of the third dose (Day 0, D0), three months after the third dose (M3) and six months post third dose (M6). Plasma was isolated through centrifugation of EDTA blood samples at 1300g for 10 mins and stored at -80°C until further use. Hemolyzed samples were excluded from the study.

### Olink proteomic analysis

Proteomic profiling of plasma samples was performed using four distinct Target 96-plex immunoassays (Olink, Sweden) covering 368 proteins, including Cardiovascular II (v.5006), Cardiovascular III (v.6113), Immune Response (v.3204), and Inflammation (v.3023). Samples and controls were randomized across two plates per panel (eight plates in total). No plate-driven batch effects were observed based on principal component analysis (PCA), although the presence of subtle batch effects cannot be completely excluded (**Supplementary Figure 1**). Quality control and data normalization of the proximity extension assay data were performed using the Olink Normalized Protein eXpression (NPX) Manager software (v3.3.2.434) and NPX values, arbitrary log2-scale values, were used to determine changes in circulating protein expression levels. Intra-assay coefficients of variation (CV) ranged from 5-9% and inter-assay CVs reached 8-12% across all panels, with interplate control inter-assay CVs of 3-7% across all panels (**Supplementary Table 1**). Outlier identification was assessed with OlinkAnalyze R package (v4.5.0) using PCA and IQR-based QC plots. One sample, QnCov-032-3P (M6 sample from recently infected individual), was identified as an outlier across all Olink panels and was excluded from downstream analyses. Proteins with expression levels below the assay-specific limit of detection (LOD) in more than 20% of samples were excluded from analysis (n=54). Sixteen proteins with values below the LOD in 5-20% of samples were imputed using the missForest algorithm (v1.5).

### Differential and longitudinal plasma proteome analysis

Following quality control and LOD filtering, 314 proteins were subjected to differential expression analysis using the LIMMA (Linear Models for MicroArray data v3.62.2) package in R programming environment (v4.4.0) (35). Pairwise comparisons were performed between Day 0, and 3- or 6-months post-booster using LIMMA moderated t-statistics, and plate identity was not included as a covariate. Differentially expressed proteins were identified based on an absolute fold change ≥ 1.25 (abs log2 fold change ≥ 0.3219) and a p-value ≤ 0.05. Benjamini–Hochberg (BH)–adjusted false discovery rate p-values (pFDR) were reported alongside unadjusted p-values (**Supplementary Table 2**). Volcano plots were generated using the ggplot2 R package (v3.5.2), and GraphPad Prism (v10.5.0) was used to generate scatter plots of the differentially expressed proteins. A Venn diagram was generated to identify proteins commonly dysregulated between infection-naïve and previously infected individuals across multiple timepoints using the ggVennDiagram R package (v1.5.4). Pearson correlation analyses between proteins were performed using corrr R package (v0.4.5) and visualized using ggcorrplot R package (v0.1.4.1). The linear mixed-effects model (LME) from the lme4 R package (v1.1-37) was used to assess longitudinal proteomic changes while accounting for within-subject correlation arising from repeated measurements (35). Timepoint (to assess temporal changes after booster vaccination), infection group (infection-naïve vs previously infected), and their interaction (time × infection group, to determine whether infection status modulated temporal protein changes) were included as fixed effects, with subject identifiers specified as a random intercept. Statistical significance of fixed effects was assessed using Type III analysis of variance (ANOVA) F-tests with Satterthwaite’s approximation for degrees of freedom via the lmerTest R package (v3.1-3) (36). P-values were adjusted for multiple testing using the Benjamini–Hochberg false discovery rate method, with pFDR ≤ 0.05 considered statistically significant.

### Elastic net regression analysis

Elastic net regression with grouped 10-fold cross-validation was conducted to explore the performance of plasma protein levels in classifying post-vaccination timepoints using glmnet R package (v4.1-10). Cross-validation folds were performed by grouping by individual, preventing information leakage, and ensuring all samples from the same individual were grouped within the same fold. Models were generated separately for infection-naïve and previously infected individuals. The optimal penalization alpha was selected via grid search (0-1) and the regularization parameter Λ was determined through lambda.1se selection. To prevent data leakage, standardization was implemented within the cross-validation framework. Model performance was evaluated using F1 score, accuracy, and area under the receiver operating characteristic curve (AUC) with 95% confidence intervals derived from 1,000 bootstrap iterations. Feature stability was quantified by calculating the frequency of protein selection across cross-validation folds. Permutation importance was calculated by randomly shuffling each selected protein 10 times and measuring the mean decrease in AUC (Δ AUC=AUC – AUC_shuffled_).

### Pathway enrichment and Gene Ontology analysis

Exploratory pathway enrichment and gene ontology (GO) analysis were performed using the Enrichr platform with the tested proteins as background (28–30), and combined scores and p-values were extracted for KEGG 2026 pathways and GO biological processes 2025. The top 20 KEGG pathways with the highest combined scores, integrating enrichment p-values and z-scores, were selected to visualize the most relevant biological pathways using the ggplot2 R package (v3.5.2). GO biological processes with p-values ≤ 0.05 were depicted in a heatmap using semantic similarity and clustering to reduce redundancy and visualize relationships between enriched terms while grouping functionally related GO terms. GO semantic similarity analysis was performed with the Wang method in the GOSemSim R package (v2.32.0) (31), and heatmaps were generated using the ComplexHeatmap R package (v2.22.0). Protein-protein interactions were visualized using STRING database.

## Results

### Circulating proteome profiles in infection-naïve individuals following booster vaccination

**Figure 2:**
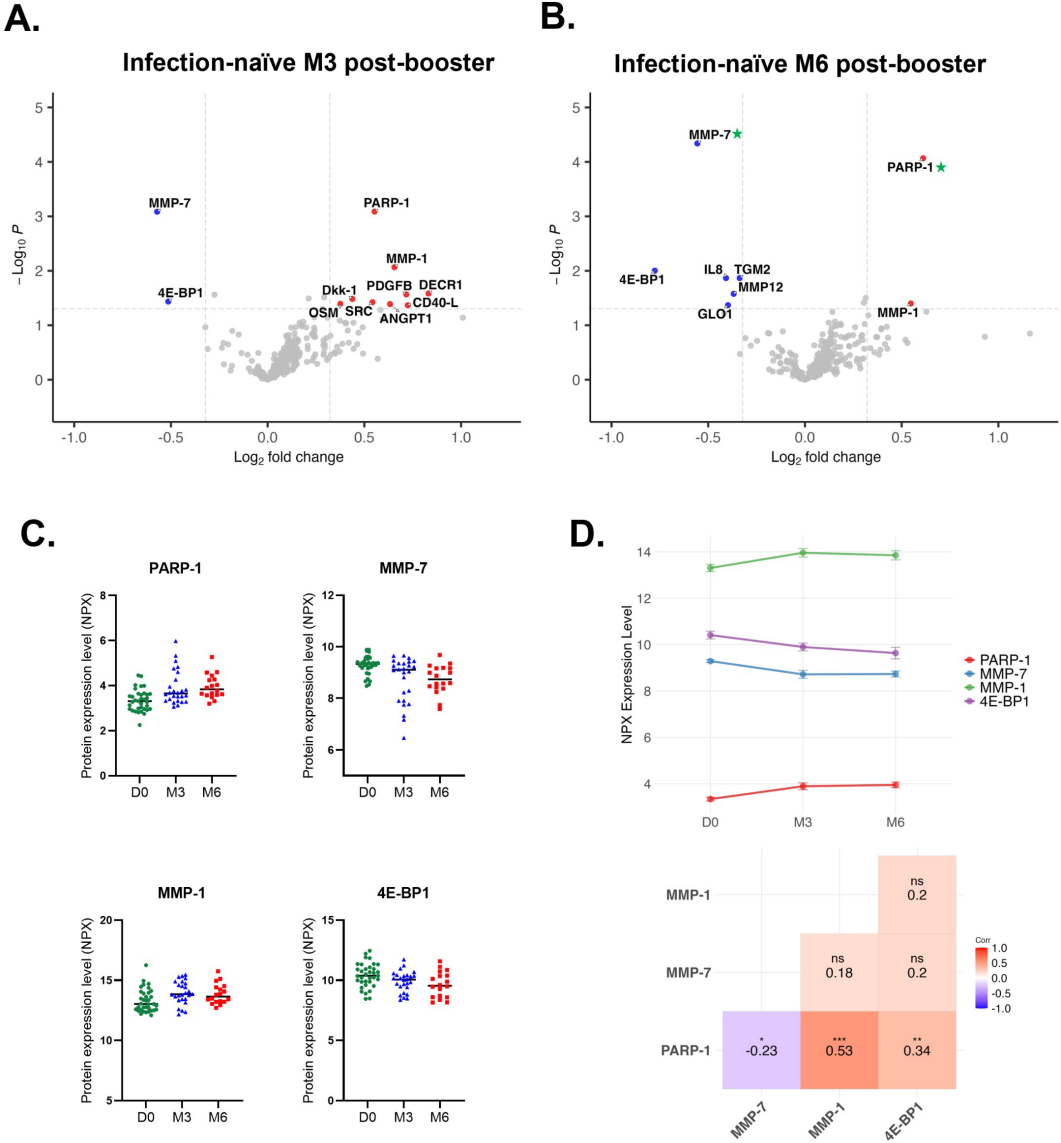
Plasma proteome alterations following third dose (booster) vaccination in infection-naïve individuals. Volcano plot (p ≤ 0.05, abs log2FC≥0.3219 or abs FC ≥ 1.25) analysis of differentially expressed proteins using LIMMA at **(A)** 3-months (M3, n=26) and **(B)** 6-months (M6, n=18) post-booster compared to D0 (n=35). Proteins highlighted in blue and red are down- and upregulated, respectively. Green asterisks indicate proteins that are significantly dysregulated with pFDR ≤ 0.05. **(C)** Scatter plots of sustained differentially expressed proteins in infection-naïve individuals at different timepoints (n=35 at D0, n=26 at M3, n=18 at M6). **(D)** Line plot of relative protein expression (mean ± SE) over time and Pearson correlation matrix of sustained differentially expressed proteins. D0, Day 0; M3, 3-month post-booster; M6, 6-months post-booster. ns, non-significant; *p ≤ 0.05; **p ≤ 0.01; ***p ≤ 0.001.

To investigate changes in the circulating proteome following BNT162b2 booster vaccination without bias from naturally acquired immunity, we determined the plasma levels of 314 proteins in infection-naïve, healthy adults (n=35) immediately prior to (D0), three months (M3) and six months (M6) after the third vaccine dose. At 3-months post-booster, we found increased circulating levels of nine proteins and a decrease in MMP-7 and 4E-BP1 levels (abs FC≥1.25, p ≤ 0.05, pFDR>0.05) (**Figure 2A**). Exploratory KEGG pathway analysis indicated these eleven proteins may be involved in multiple signaling pathways, gap/adherents/tight cell junctions, choline metabolism, bacterial and viral infections, and inflammatory mediator regulation of Transient Receptor Potential (TRP) channels (**Supplementary Figure 2A**). Gene ontology analysis revealed five clusters of enriched processes related to cardiac muscle hypertrophy, cardiac function, protein localization to chromatin and chromosomes, negative regulation of protein binding, negative regulation of protein-containing complex assembly and telomere maintenance, however, these results warrant further investigation in future studies (**Supplementary Figure 2B**). At 6-months post-booster, PARP-1 (FC = 1.53, p=8.59x10^-5^, pFDR=0.01) and MMP-7 (FC = 0.68, p=4.58x10^-5^, pFDR=0.01) were significantly up- and downregulated, respectively, while the levels of MMP-1 (FC = 1.46, p=0.04, pFDR>0.05) and five other proteins (p ≤ 0.05, pFDR>0.05) were altered but did not reach significance under pFDR (**Figure 2B**). Together, the differentially expressed proteins at 6-months post-booster are predicted to be involved in similar signaling pathways as those observed at 3-months post-booster in addition to cellular senescence, multiple immune response signaling pathways (NF-κB, PPAR, RIG-I-like, phospholipase D, IL-17), and viral infections (**Supplementary Figure 3A**). Gene ontology analysis further highlighted potential dysregulation of calcium homeostasis, extracellular matrix assembly and structure organization, and protein localization to chromatin and chromosomes (**Supplementary Figure 3B**). Notably, early changes in circulating levels of PARP-1, MMP-1, MMP-7, and 4E-BP1 persisted up to six months after receiving the third dose, with MMP-1 showing the highest expression levels followed by 4E-BP1, MMP-7 and PARP-1 (**Figures 2C, D**). Correlation analysis revealed moderate-to-strong positive correlations between PARP-1 and MMP-1 levels (r=0.53), and PARP-1 and 4E-BP1 levels (r=0.34) (**Figure 2D**). In contrast, PARP-1 levels exhibited a weak negative correlation with MMP-7 (r=-0.23).

### Circulating proteome profiles in previously infected individuals following booster vaccination

**Figure 3:**
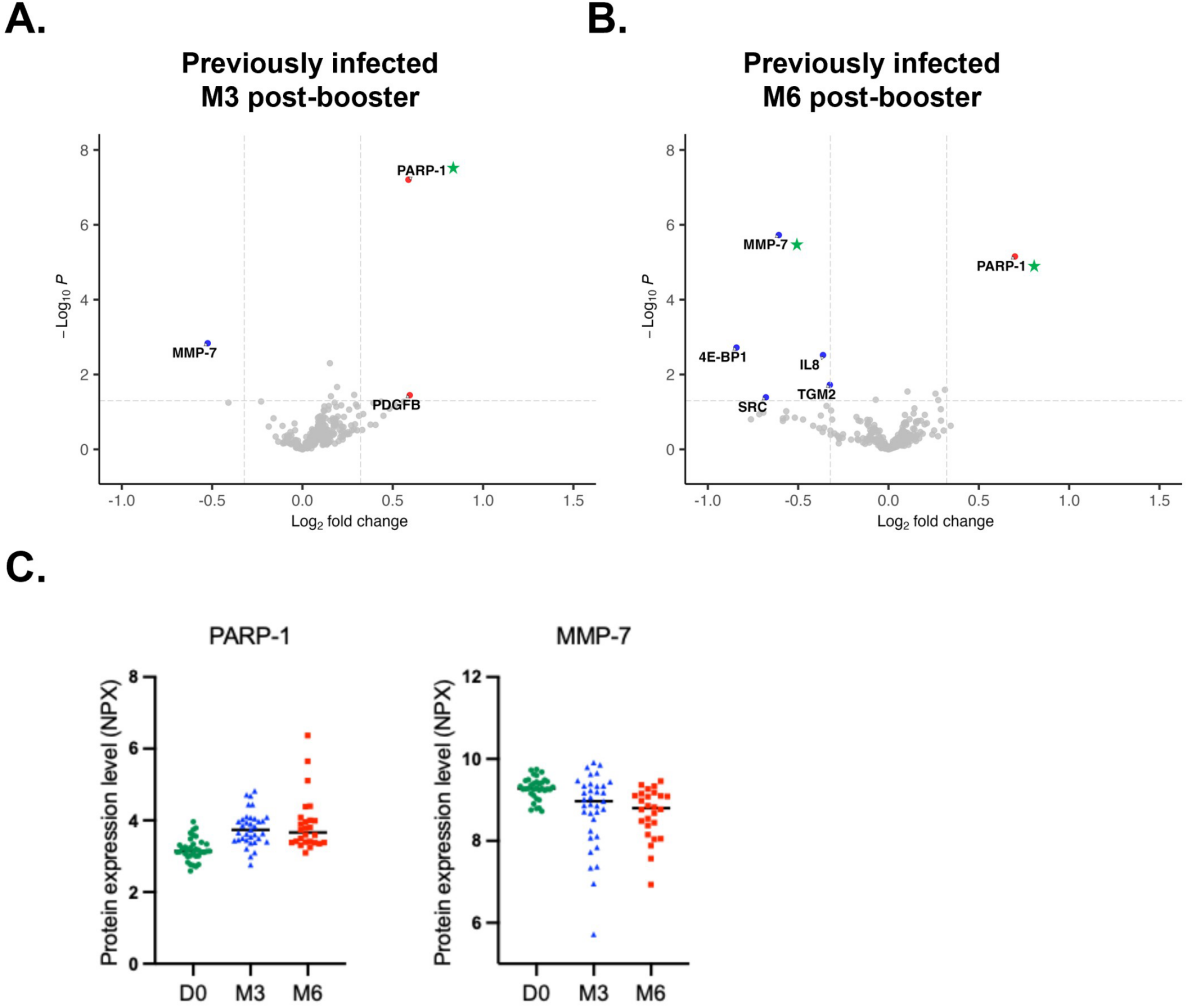
Plasma proteome alterations following third dose (booster) vaccination in previously infected individuals. Volcano plot (p ≤ 0.05, abs log2FC≥0.3219 or abs FC ≥ 1.25) of differentially expressed proteins using LIMMA at **(A)** 3-months (n=35) and **(B)** 6-months (n=26) post-booster compared to D0 (n=35). Proteins highlighted in blue and red are down- and upregulated, respectively. Green asterisks indicate proteins that are significantly dysregulated with pFDR≤ 0.05. **(C)** Scatter plots of sustained differentially expressed proteins PARP-1 and MMP-7 at different timepoints. D0, Day 0; M3, 3-month post-booster; M6, 6-months post-booster.

Next, we assessed whether individuals with prior SARS-CoV-2 infection (n=35) – characterized by higher anti-spike, anti-RBD and anti-nucleocapsid IgG antibody levels (27) - exhibit distinct vaccine-induced changes in the circulating proteome. At 3-months post-booster, PARP-1 levels were significantly increased compared to pre-booster levels (FC = 1.5, p=6.22x10^-8^, pFDR=1.95 x10^-5^) whereas MMP-7 levels were decreased (FC = 0.69, p=0.001, pFDR>0.05) and PDGFB levels were increased (FC = 1.51, p=0.03, pFDR>0.05) (**Figure 3A**). At 6-months, we observed a significant decrease in MMP-7 (FC = 0.66, p=1.86x10^-6^, pFDR=5.8x10^-4^) alongside a significant increase in PARP-1 (FC = 1.62, p=7.0x10^-6^, pFDR=0.001), and decreased levels of 4E-BP1 (FC = 0.56, p=1.9x10^-3^, pFDR>0.05), IL8 (FC = 0.78, p=3x10^-3^, pFDR>0.05), TGM2 (FC = 0.80, p=0.002, pFDR>0.05), and SRC (FC = 0.62, p=0.04, pFDR>0.05) (**Figure 3B**). PARP-1 levels exhibited a persistent increase, whereas MMP-7 levels showed a sustained decrease over time (**Figure 3C**). KEGG pathway analysis at 6-months post-booster revealed pathway enrichment patterns similar to those in infection-naïve individuals with the addition of pathways related to adherens and tight junctions, inflammatory mediator regulation of TRP channels, and various bacterial and viral infection processes (**Supplementary Figure 4A**). GO analysis highlighted clusters of processes related to cardiac muscle hypertrophy and function, calcium homeostasis, cell cycle regulation, protein localization to chromatin and chromosomes, some which were also observed in infection-naïve individuals and require further investigation to support these exploratory findings (**Supplementary Figure 4B**).

### Booster vaccination induces a robust and consistent alteration of circulating MMP-7 and PARP-1 levels irrespective of prior SARS-CoV-2 infection

**Figure 4:**
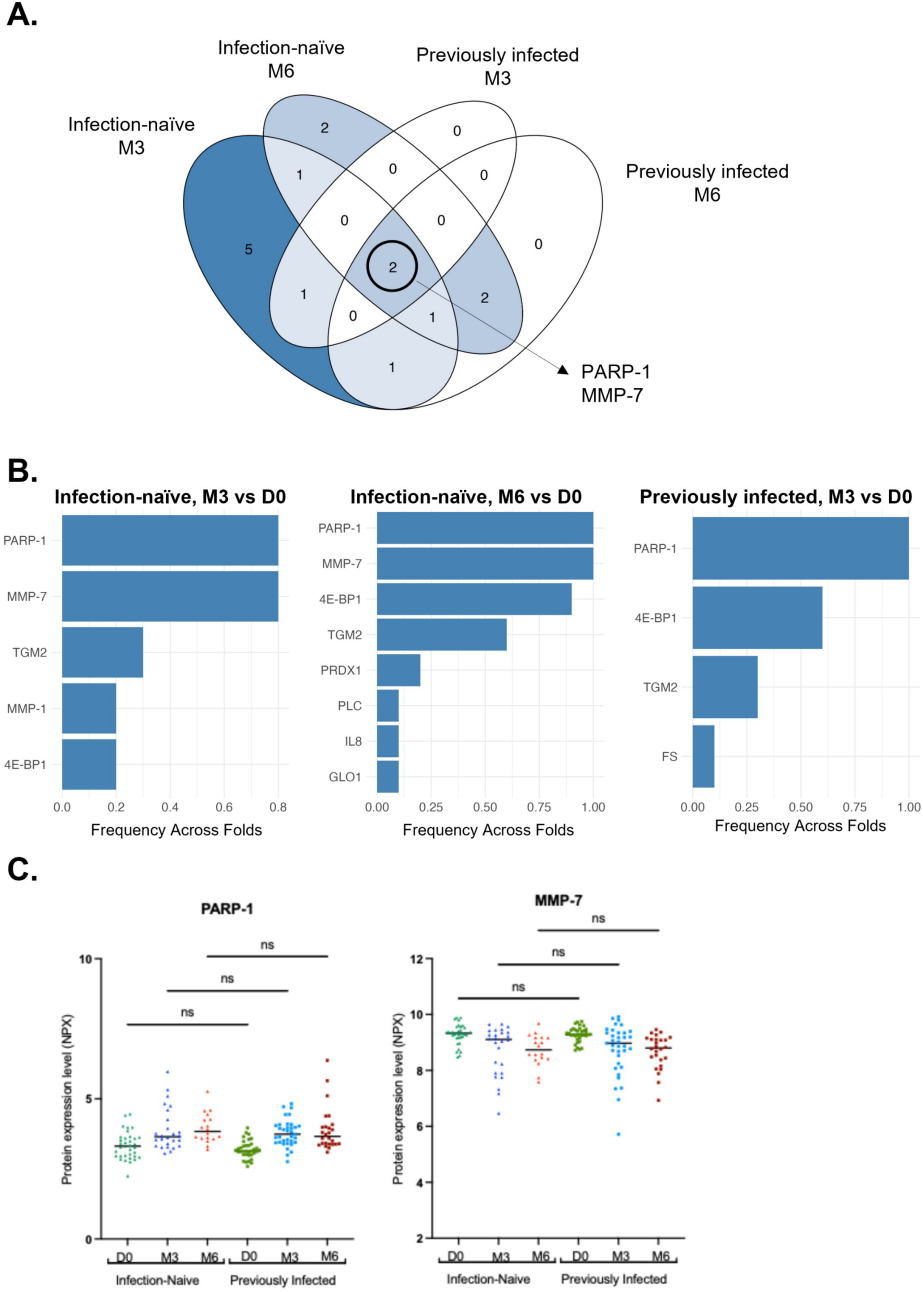
Sustained plasma proteome alterations, irrespective of prior infection status. **(A)** Venn diagram depicting the differentially expressed proteins in infection-naïve and previously infected individuals across timepoints as determined by LIMMA. **(B)** Feature selection frequency of exploratory elastic net regression. Bar plots show how frequently each protein was selected at M3 versus D0 and M6 versus D0 in infection-naïve, and at M3 versus D0 in previously infected individuals. Bar plot for feature selection frequency at M6 versus D0 in previously infected individuals is not represented as all 314 proteins were selected. **(C)** Scatter plots of PARP-1 and MMP-7 as sustained dysregulated circulating proteins in both infection naïve and previously infected individuals at D0 (n=35 for infection-naïve and previously infected), M3 (infection naïve, n=26; previously infected, n=34) and M6 (infection-naïve, n=18; previously infected, n=26). D0, Day 0; M3, 3-month post-booster; M6, 6-months post-booster. ns, non-significant.

Comparative analysis of infection-naïve and previously infected individuals identified MMP-7 and PARP-1 levels to be consistently altered at both 3- and 6- months following the third vaccine dose administration (**Figure 4A**). Given the relatively small sample size of our study, exploratory elastic net regression was applied as a multivariate pattern recognition approach to independently evaluate our differential expression findings (**Supplementary Table 3**). Feature stability analysis identified PARP-1 and MMP-7 as the most consistently selected proteins in infection-naïve individuals at 3-months and 6-months post-booster. In addition, both proteins were associated with the highest permutation importance scores (**Supplementary Figure 5**). In previously infected individuals, PARP-1, but not MMP-7, was identified as a consistently selected protein at 3-months post-booster only, likely due to the small sample size negatively impacting the power of the analysis (**Figure 4B**).

Circulating PARP-1 levels gradually increased over time in both infection-naïve and previously infected individuals, while MMP-7 levels demonstrated a clear stepwise decrease over time in both groups (**Figure 4C**). Of note, no significant differences were found between infection-naïve and previously infected individuals at any timepoint, suggesting that the longitudinal changes in these proteins are primarily driven by the booster vaccination itself. To complement the LIMMA analysis and explicitly account for within-subject variability across repeated measurements, we applied linear mixed-effects modeling across all proteins. This analysis identified 19 proteins exhibiting significant temporal changes (time effect, FDR p-value ≤ 0.05, **Supplementary Table 4**), including PARP-1 and MMP-7. In contrast, no proteins showed significant effects of infection group, or time × infection group interactions. Furthermore, the PARP-1 and MMP-7 longitudinal expression patterns were retained in linear mixed-effect sensitivity analyses when participants with a medical history were excluded (**Table 1**, n=6, data not shown). Collectively, these findings indicate that infection-naïve and previously infected individuals exhibit comparable longitudinal proteomic changes in response to booster vaccination.

### Post-booster circulating proteome profiles in previously infected individuals vary according to the time elapsed since infection

**Figure 5:**
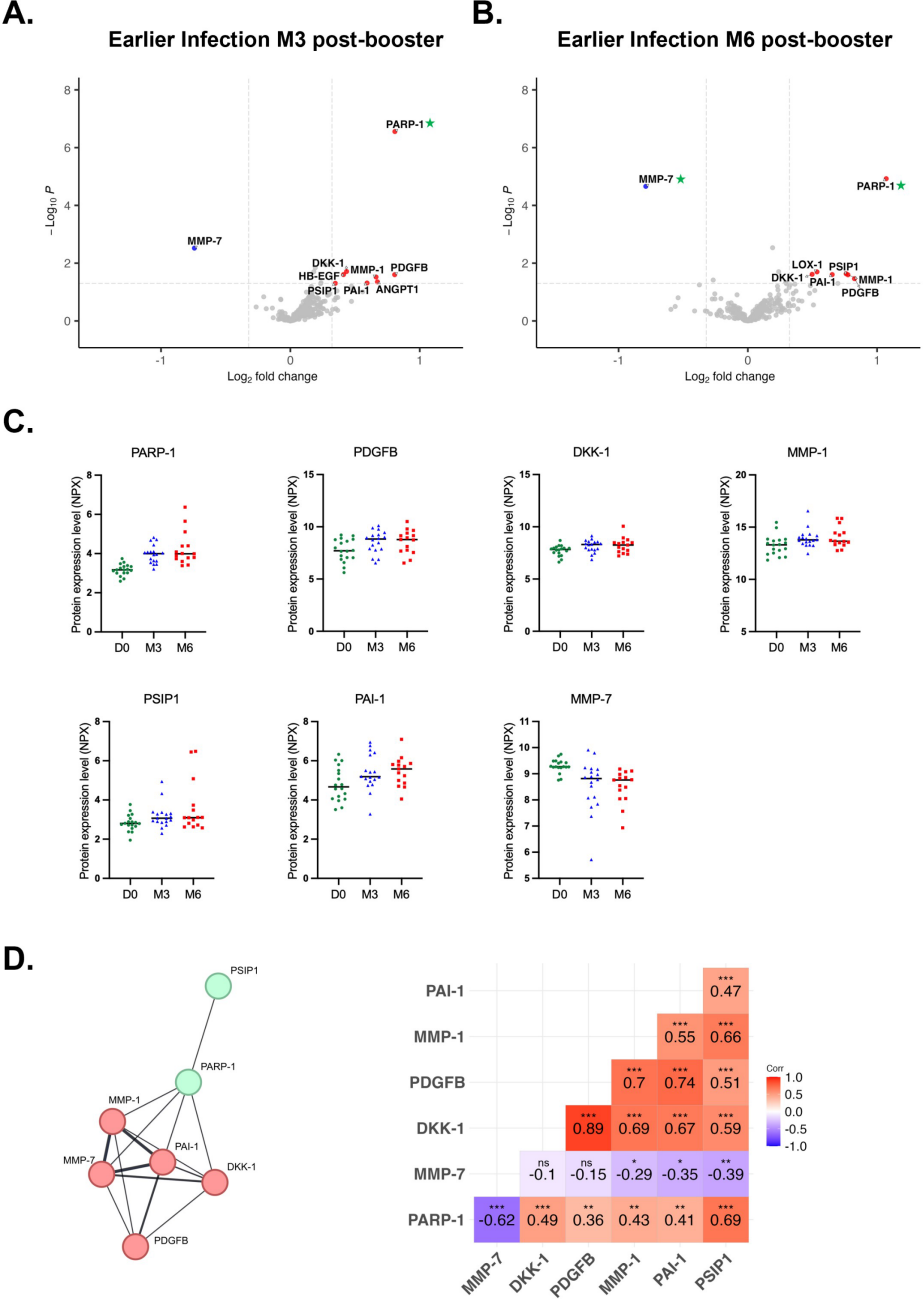
Plasma proteome alterations following third dose (booster) vaccination in individuals with earlier infections (before the second vaccine dose). Volcano plots of differentially expressed proteins (p ≤ 0.05, abs log2FC≥0.3219 or abs FC ≥ 1.25) using LIMMA at **(A)** 3-months (n=18) and **(B)** 6-months (n=15) post-booster compared to D0 (n=18). Proteins highlighted in blue and red are down- and upregulated, respectively. Green asterisks indicate proteins that are significantly dysregulated with pFDR ≤ 0.05. **(C)** Scatter plots of seven common dysregulated proteins at 3- and 6-months post-booster. **(D)** Protein-protein interaction (PPI) network and Pearson correlation matrix of the seven common differentially expressed proteins. PPI clusters are highlighted in red and green with the thickness of the lines between proteins indicating the predicted degree of confidence of the interactions. ns, non-significant, *p ≤ 0.05; **p ≤ 0.01, ***p ≤ 0.001.

**Figure 6:**
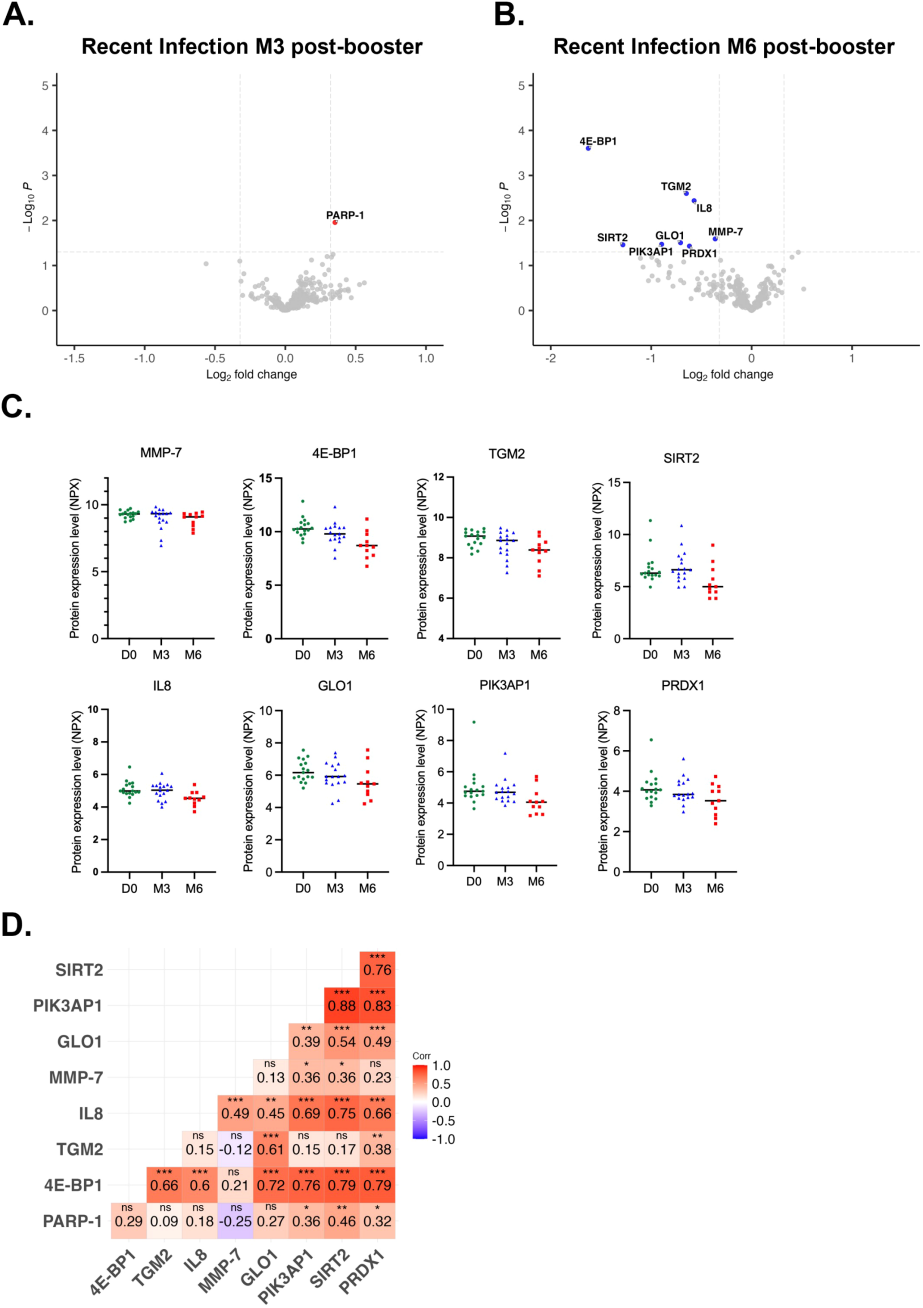
Plasma proteome alterations following third dose (booster) vaccination in individuals with recent infections (after the second vaccine dose). Volcano plots of differentially expressed proteins (p ≤ 0.05, abs log2FC≥0.3219 or abs FC ≥ 1.25) using LIMMA at **(A)** 3-months (n=17) and **(B)** 6-months (n=11) post-booster compared to D0 (n=17). Proteins highlighted in blue and red are down- and upregulated, respectively. Green asterisks indicate proteins that are significantly dysregulated with pFDR≤ 0.05. **(C)** Scatter plots of differentially expressed proteins at 6-months post-booster. **(D)** Pearson correlation matrix of the nine differentially expressed proteins post-booster. D0, Day 0; M3, 3-month post-booster; M6, 6-months post-booster. ns, non-significant; *p ≤ 0.05; **p ≤ 0.01; ***p ≤ 0.001.

To investigate how prior SARS-CoV-2 infection may affect the post-booster circulating proteome, we compared the expression profiles of individuals with recent infections (after completion of the two-dose regimen, n=17) and those with earlier infections (before receiving the second dose, n=18). Individuals with earlier infections displayed a broad range of upregulated circulating protein levels. At 3-months post-booster, PARP-1 levels were significantly upregulated (FC = 1.75, p=2.77x10^-7^, pFDR=8.7x10^-5^), in addition to elevated levels of seven other proteins (abs FC ≥ 1.25, p ≤ 0.05, pFDR>0.05) and reduced levels of MMP-7 (FC = 0.6, p=0.003, pFDR>0.05) (**Figure 5A**). Likewise, at 6-months post-booster we observed a significant downregulation of MMP-7 (FC = 0.58, p=2.2x10^-5^, pFDR=0.003) and significant upregulation of PARP-1 (FC = 2.1, p=1.2x10^-5^, pFDR=0.003). In addition, six out of eight proteins that were upregulated at 3-months post-booster were also elevated at 6-months post-booster in addition to increased levels of LOX-1 (FC = 1.4, p=0.02, pFDR>0.05) (**Figure 5B**). When comparing their expression levels over time, we observe consistent expression of the six common upregulated proteins across timepoints, whereas MMP-7 levels clearly decrease over time (**Figure 5C**). KEGG pathway analysis (**Supplementary Figure 6A**) of these seven common dysregulated proteins predicted enrichment of immune-related signaling pathways such as Wnt signaling, Apelin, p53, Hippo, phospholipase D, PPAR, alongside viral life cycle of HIV-1, and cellular senescence, suggesting potential activation of anti-viral immune responses. GO analyses demonstrated potential enrichment in biological processes related to Wnt signaling, regulation of protein binding, protein localization to chromatin and chromosomes, and extracellular matrix disassembly and structure organization (**Supplementary Figure 6B**). PPI analysis revealed two clusters (**Figure 5D**). Cluster 1 (red) forms a high-confidence network comprising MMP-1, MMP-7, and PAI-1, alongside DKK-1 and PDGFB. Within this cluster, DKK-1 levels were strongly correlated with PDGFB (r=0.89), MMP-1 (r=0.69), and PAI-1 (r=0.67), while PDGFB levels correlated strongly with MMP-1 (r=0.70) and PAI-1 (r=0.74) (**Figure 5D**). In cluster 2 (green), PARP-1 and PSIP1 exhibited a moderate correlation in expression levels (r=0.69). In contrast, individuals with recent infections demonstrated fewer upregulated, but more downregulated, blood proteomic changes. Recent exposure to SARS-CoV-2 was associated with increased levels of PARP-1 (FC = 1.28, p=0.01, pFDR>0.05) at 3-months post-booster (**Figure 6A**). At 6-months post-booster, expression of MMP-7 (FC = 0.77, p=0.02, pFDR>0.05), 4E-BP1 (FC = 0.56, p=1.9x10^-3^, pFDR>0.05), TGM2 (FC = 0.64, p=2.5x10^-3^, pFDR>0.05), SIRT2 (FC = 0.41, p=0.03, pFDR>0.05), IL8 (FC = 0.68, p=3x10^-3^, pFDR>0.05), GLO1 (FC = 0.61, p=0.03, pFDR>0.05), PIK3AP1 (FC = 0.54, p=0.03, pFDR>0.05), and PRDX1 (FC = 0.65, p=0.04, pFDR>0.05) were reduced (**Figure 6B**), reflecting a gradual decrease over time (**Figure 6C**). Correlation analysis (**Figure 6D**) revealed strong correlation between the expression levels of 4E-BP1 and GLO1 (r=0.72), PIK3AP1 (r=0.76), SIRT2 (r=0.79) and PRDX1 (r=0.79), as well as between SIRT2 and PIK3AP1 (r=0.88), IL8 (r=0.75) and PRDX1 (r=0.76), and between PIK3AP1 and PRDX1 (r=0.83).

Exploratory KEGG pathway analysis identified enrichment of pathways related to cellular senescence, metabolism, various signaling pathways including immune-related signaling pathways, bacterial and viral infections (**Supplementary Figure 7A**). GO analysis indicated the observed proteomic changes to be potentially involved in responses to various stimuli, protein deacylation, heterochromatin formation, fatty acid metabolism, epigenetic regulation, calcium homeostasis, myelination, mitotic nuclear organization, and immune cell activation (**Supplementary Figure 7B**).

## Discussion

To better understand booster-induced immune responses, we investigated the impact of administering the third dose or first booster dose of the BNT162b2 mRNA vaccine on the circulating proteome. We performed a comprehensive longitudinal plasma proteomic analysis to assess changes in circulating protein levels before and up to six months after receiving the third dose, and to determine whether prior SARS-CoV-2 infection modulates vaccination-associated proteomic changes. In infection-naïve individuals, booster vaccination was associated with increased plasma PARP-1 and MMP-1 levels whereas MMP-7 and 4E-BP1 levels showed a sustained decrease. Similarly, previously infected individuals exhibited persistent dysregulation of PARP-1 and MMP-7 following booster vaccination. These findings suggest that PARP-1 and MMP-7 plasma levels are consistently modulated following administration of the third vaccine dose, irrespective of prior infection. While expression levels of both proteins changed significantly over time, linear mixed-effects modeling demonstrated that these changes were independent of prior infection status, suggesting that the observed temporal changes were primarily vaccination-driven effects that were common across all individuals. Although our cohort size is relatively small, exploratory elastic net regression consistently identified PARP-1 and MMP-7 as top selected proteins in infection-naïve individuals at both 3- and 6-months post-booster, together with PARP-1 in previously infected individuals at 3-months post-booster. While machine learning approaches are widely applied for predictive modeling, they can also capture complex multivariate patterns that may elude classical differential expression analysis. Although TGM2 and 4E-BP1 were not differentially expressed across all timepoints in infection-naïve and previously infected individuals, elastic net regression consistently selected these proteins in infection-naïve individuals at both time points and in previously infected individuals at 6-months post-booster. However, given the small cohort size we were not able to apply class imbalance correction or nested cross-validation for hyperparameter tuning, which should be performed in future independent cohorts. As such, these findings should be interpreted as exploratory and hypothesis-generating, suggesting that PARP-1 and MMP-7 may reflect biologically meaningful alterations in the plasma proteome associated with post-booster vaccination responses. Hence, circulating PARP-1 and MMP-7 levels may represent clinically relevant longitudinal biomarkers of response to BNT162b2 booster vaccination. Importantly, the stability of the Sars-CoV-2 mRNA vaccine is improved through N1-methyl-pseudouridine substitution of the mRNA encoding the spike protein and lipid nanoparticle encapsulation, enabling sustained spike protein production (32, 33). Detectable levels of spike protein have been observed in tissues and the circulation for several weeks and months, with one study reporting circulating spike protein levels up to nearly two years post-vaccination in individuals with post-vaccination syndrome (34–39). In addition, prolonged proteomic changes may also result from sustained inflammatory and tissue remodeling responses initiated by vaccination. Hence, longitudinal plasma proteomic changes, such as those identified in our study, could be used as minimally invasive biomarkers of vaccine-induced immunity. PARP-1 is a key regulator of chromatin remodeling and inflammatory gene transcription, and plays a key role in PARylation of viral proteins and NF-κB-mediated pro-inflammatory properties during viral infection (40–45). COVID-19 patients with severe disease and critically ill patients exhibit elevated plasma PARP-1 levels compared to patients with mild symptoms or healthy controls (46). Furthermore, COVID-19 patients carrying the rs8679 TT polymorphism, disrupting a miRNA binding site in the 3’ UTR region of PARP-1, have been reported to experience higher COVID-19 disease severity (47). Preclinical studies further support a role for PARP-1 in antiviral responses as pharmacological inhibition of PARP has been shown to interfere with SARS-CoV-2 entry and replication *in vitro* (48–50). In accordance, clinical observations indicate that cancer patients receiving PARP inhibitors exhibit reduced immunogenic responses to SARS-CoV-2 vaccination, suggesting that additional vaccination doses may be required to achieve optimal immunity in this population (51, 52). Collectively, these findings have prompted further preclinical research into repurposing PARP-1 targeting drugs and natural compounds to limit Sars-CoV-2 viral replication and ultimately mitigating PARP-1 mediated COVID-19 symptoms such as increased inflammation and oxidative stress, endothelial dysfunction, and acute lung injury (46, 53–60). In addition to the increased PARP-1 levels in our study, we observed a reduction in plasma MMP-7 levels following booster vaccination which may have immunological relevance. Anti-inflammatory M2 macrophages are known to secrete multiple MMPs, including MMP-2, MMP-7 and MMP-9, while loss of MMP-7 promotes M1 macrophage polarization and inflammation in mouse models of *H. pylori* infection (61, 62). As such, the decrease in MMP-7 levels in our study may reflect reduced M2 macrophage activation or abundance. Elevated levels of serum M2 macrophage activation markers and MMP-7 have been shown to associate with adverse outcomes in COVID-19, including acute respiratory distress syndrome, impaired pulmonary function, and prolonged recovery, supporting its role in tissue remodeling and disease severity (63–65). Furthermore, MMP-7 levels have been shown to increase in both plasma and lung tissue following SARS-CoV-2 infection, with plasma levels additionally increasing after vaccination, whereby these changes have been linked to altered expression of angiogenesis-related genes in blood and pulmonary tissue abnormalities, highlighting its context-dependent functions (57–67). Taken together, the complex, context-dependent function of MMP-7 underscores the need to integrate longitudinal proteomic profiling with immune cell phenotyping to more precisely define its role in vaccine-induced responses. We previously characterized humoral and cellular CD4+ and CD8+ T cell responses in this cohort (27), however, macrophage frequency and polarization remain to be investigated. While our findings suggest a potential involvement of PARP-1 and MMP-7 in post-vaccination immune responses, these observations should be interpreted with caution and warrant further validation in independent, larger cohorts with orthogonal quantitative approaches, cell-type specific profiling, and well-characterized clinical outcomes. Notably, individuals who acquired natural immunity earlier (before completion of two-dose vaccine regimen) demonstrated substantial upregulated proteome alterations, including a sustained downregulation of MMP-7 and upregulation of PARP-1 in addition to a persistent upregulation of MMP-1, PDGFB, DKK-1, PSIP1 and PAI-1, and may collectively affect processes associated with immune-related signaling pathways and tissue remodeling. In contrast, recently infected individuals (after completion of two-dose vaccine regimen) displayed only reduced levels of eight proteins at 6-months post-booster, including MMP-7. Future studies are needed to dissect the molecular mechanisms and biological processes associated with the observed proteomic changes in individuals with earlier infections as compared to recently infected individuals.

We observed longitudinal proteomic changes, which in exploratory KEGG pathway and GO analyses mapped to processes such as cardiac muscle hypertrophy and function, calcium homeostasis, and extracellular matrix and structure remodeling – processes whose dysregulation have been implicated in COVID-19 pathophysiology and have been linked to vaccine-associated events (66, 67). For example, integrative analysis of vaccine-induced transcriptional signatures with chemogenomic profiling demonstrated that BNT162b2 vaccination modulates calcium homeostasis, a pathway previously associated with commonly reported post-vaccination symptoms such as headaches, fatigue, nausea, and, in rare cases, myocarditis or myopathies including cardiomyopathy (68, 69). While these pathway-level associations provide biological context for our observed proteomic changes, the study was not designed to assess clinical outcomes, and further studies integrating molecular profiling with detailed clinical phenotyping will be important to better define their functional and clinical significance. For example, clinical follow-up data on long COVID-related symptoms, such as neurological sequelae, will be required to determine whether the observed proteomic changes translate into clinically meaningful outcomes (70–75). Moreover, as this study was conducted in a predominantly male cohort of craft and manual workers in Qatar, validation in larger and more diverse population-based cohorts will be critical to establish the generalizability and external validity of our findings. Our findings could be further strengthened by proteomic profiling of participants who did not receive a third dose, which would help delineate vaccination-specific effects from potential environmental factors affecting participants irrespective of vaccination status. Finally, it is important to acknowledge that our study did not account for differences in infection severity, circulating variant epoch or existing immunity against prevalent microbial pathogens as potential determinants of proteomic changes (76).

In summary, this longitudinal proteomic analysis provides key insights into the dynamic biological responses elicited by BNT162b2 mRNA booster vaccination, highlighting how specific proteins and pathways are modulated over time in both infection-naïve and previously infected individuals. Our findings suggest that vaccine responsiveness depends on activating robust primary immune responses in infection-naïve individuals and re-engaging inflammatory and translational programs in previously infected individuals. The consistent dysregulation of PARP-1 and MMP-7 across all individuals underscores their central role in post-vaccination immune responses.

## Data Availability

The raw data supporting the conclusions of this article will be made available by the authors, without undue reservation.
