# Supplementary figures and images for: Post-booster longitudinal plasma proteomic changes following BNT162b2 COVID-19 vaccination in Qatar
Source: Front Immunol. 2026 Apr 24;17:1762522. doi: 10.3389/fimmu.2026.1762522 (PMC13152856; doi:10.3389/fimmu.2026.1762522)

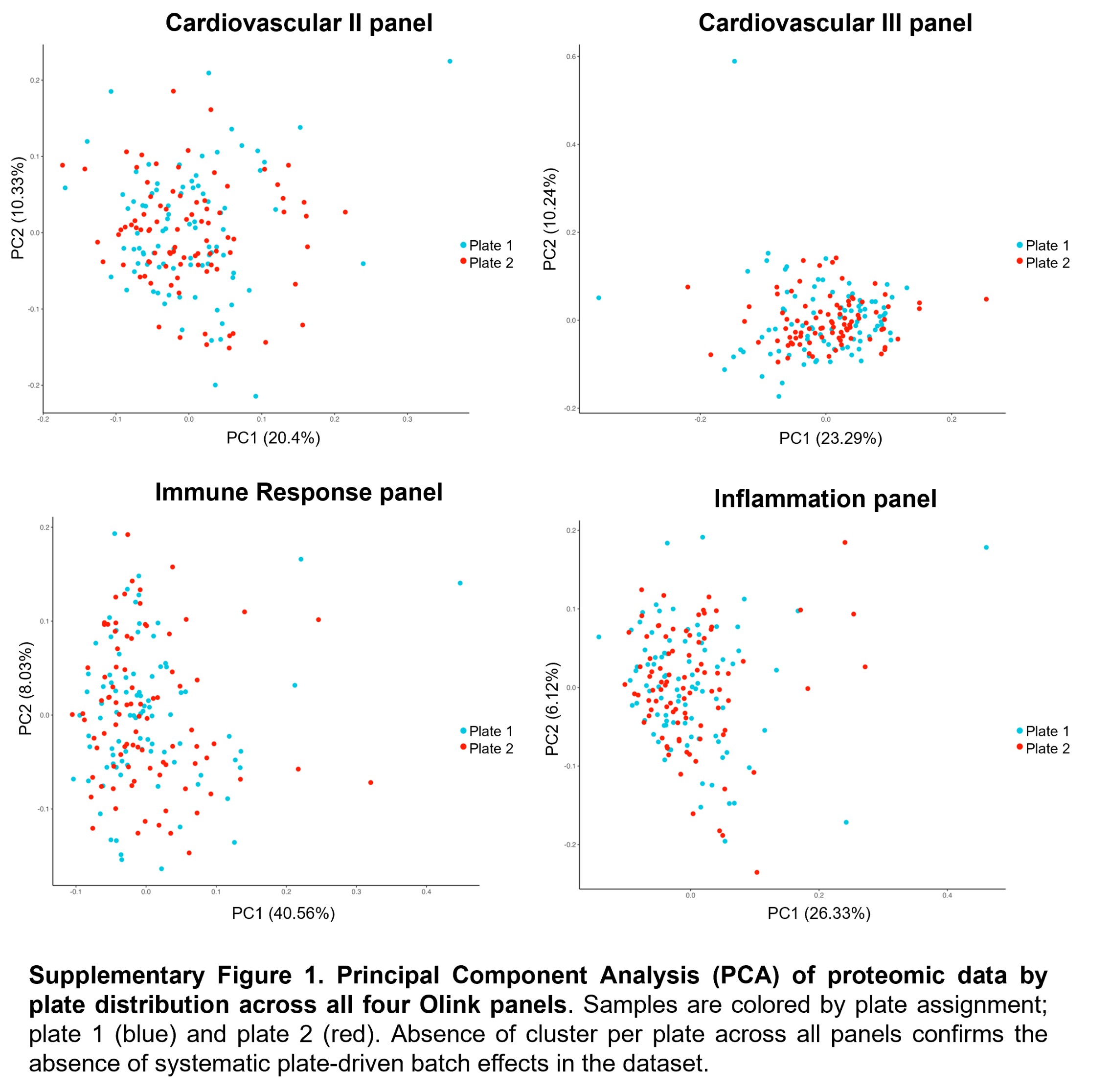

Supplement: Supplementary file 1 [file Image1.jpeg]

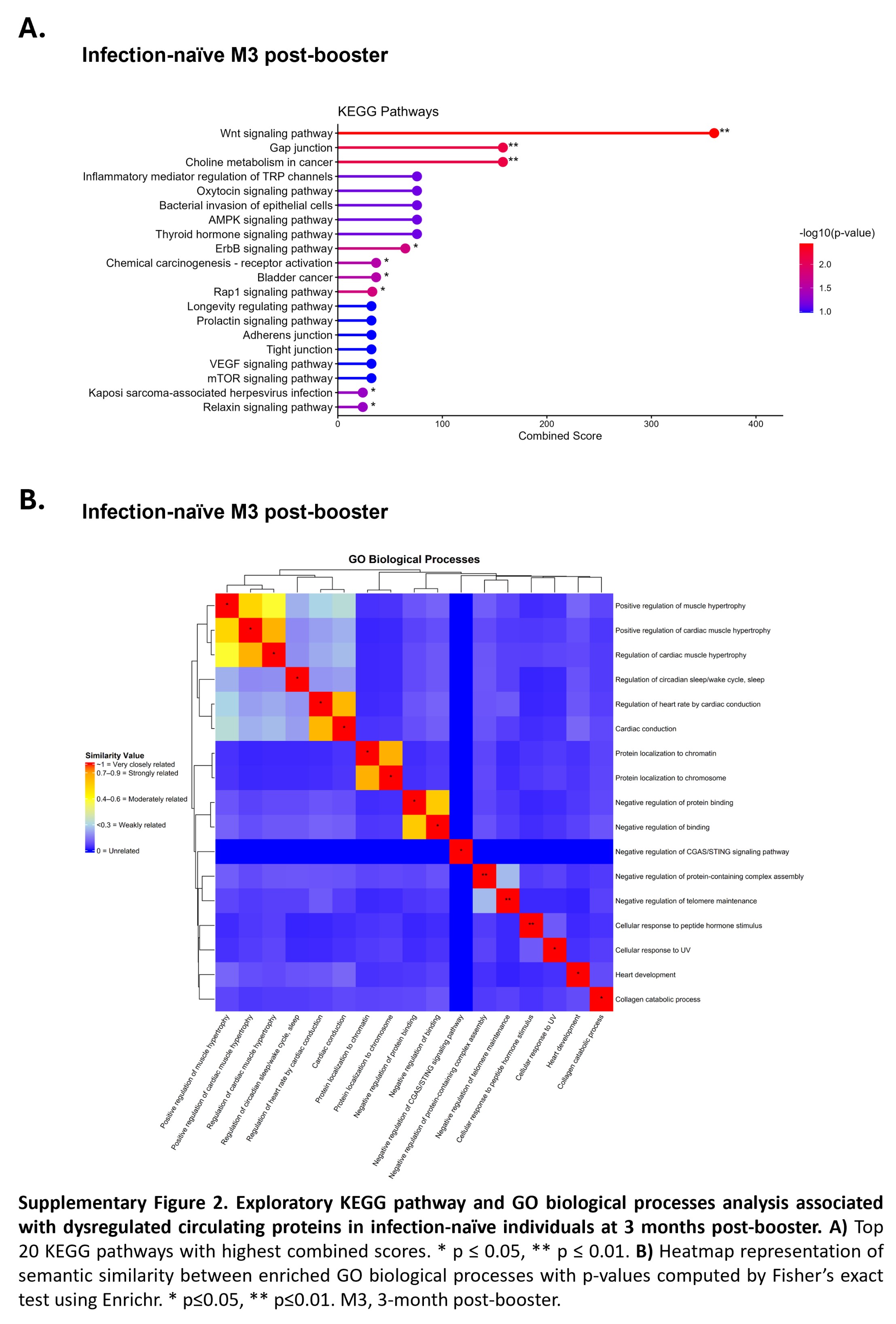

Supplement: Supplementary file 2 [file Image2.jpeg]

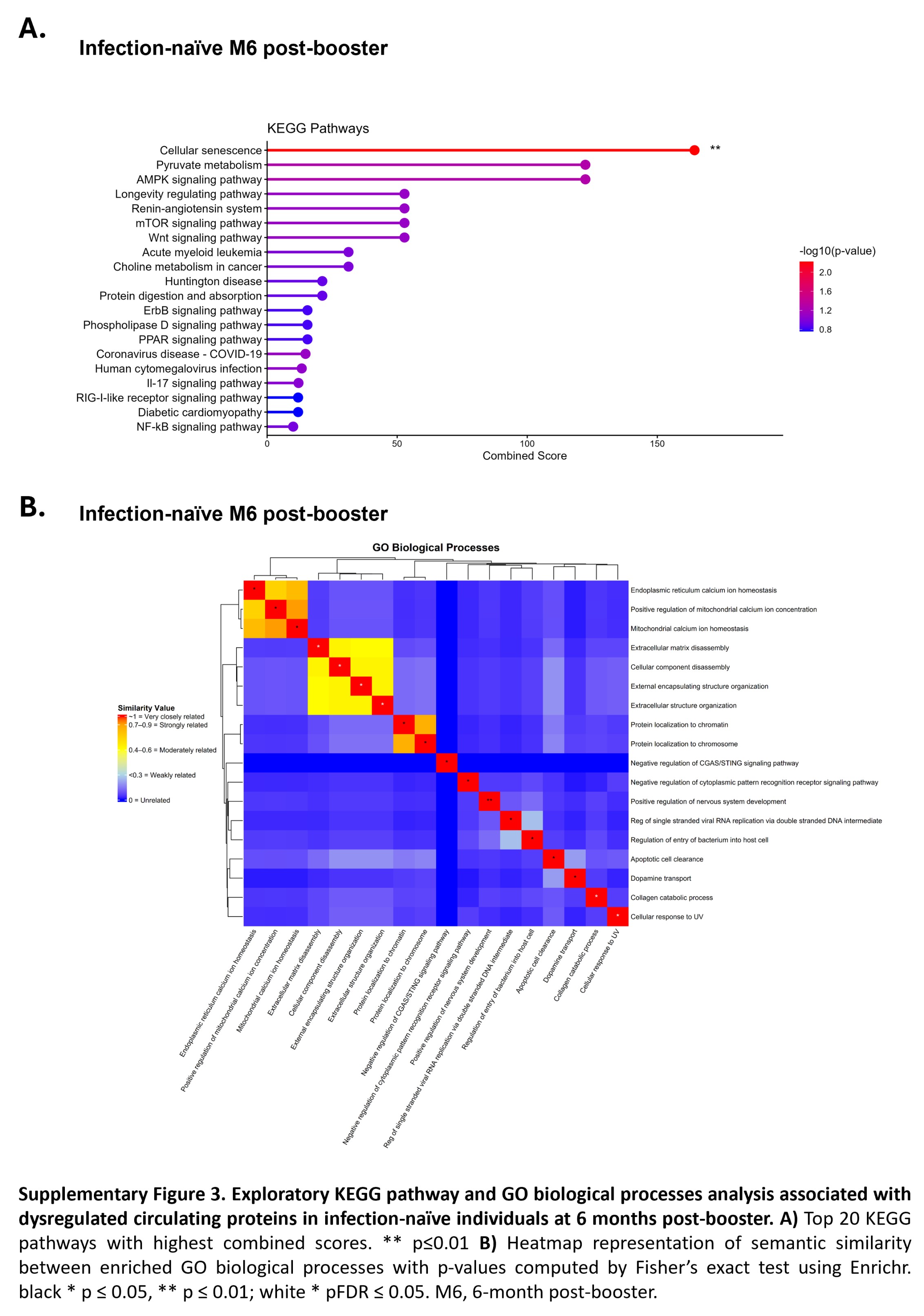

Supplement: Supplementary file 3 [file Image3.jpeg]

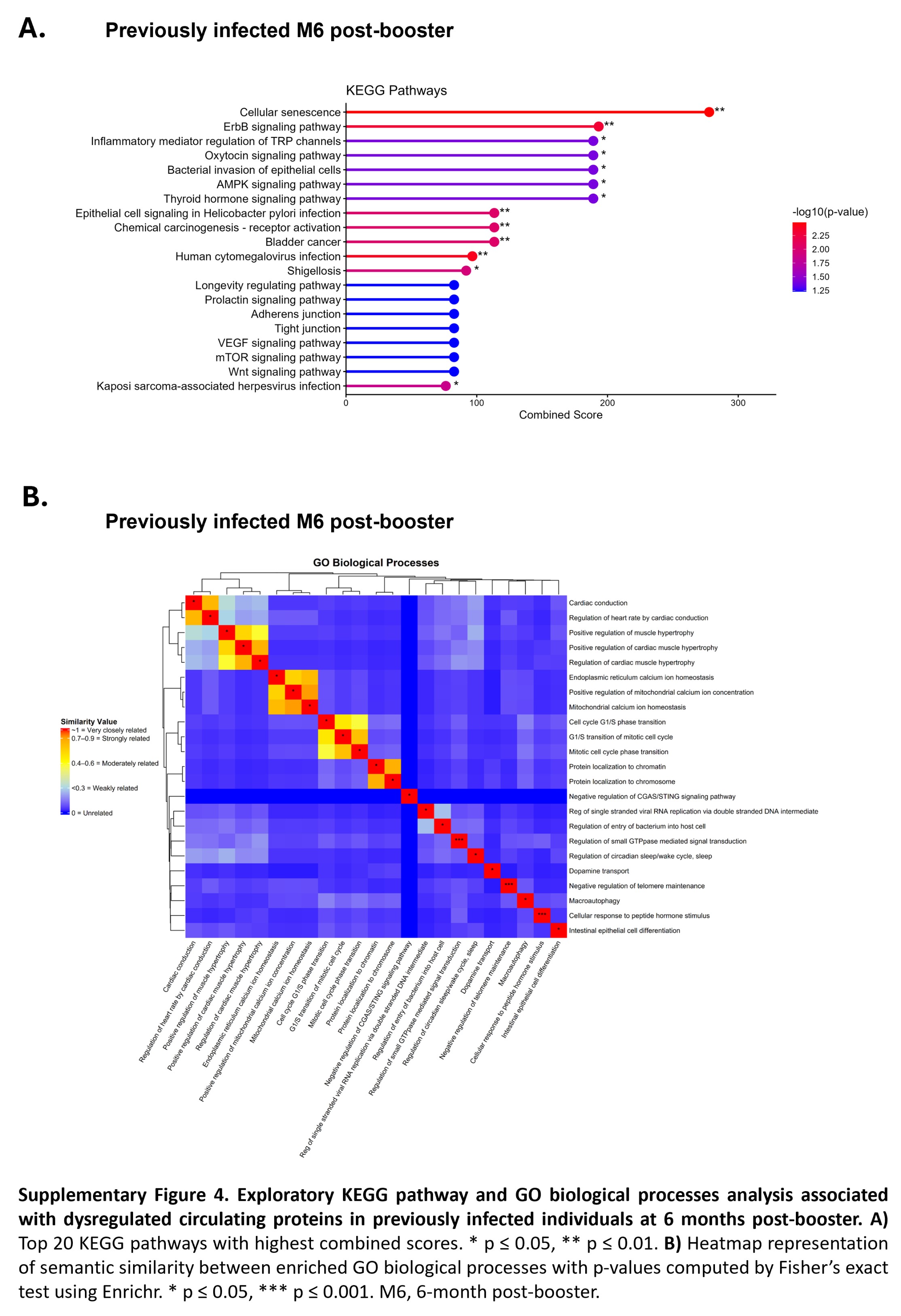

Supplement: Supplementary file 4 [file Image4.jpeg]

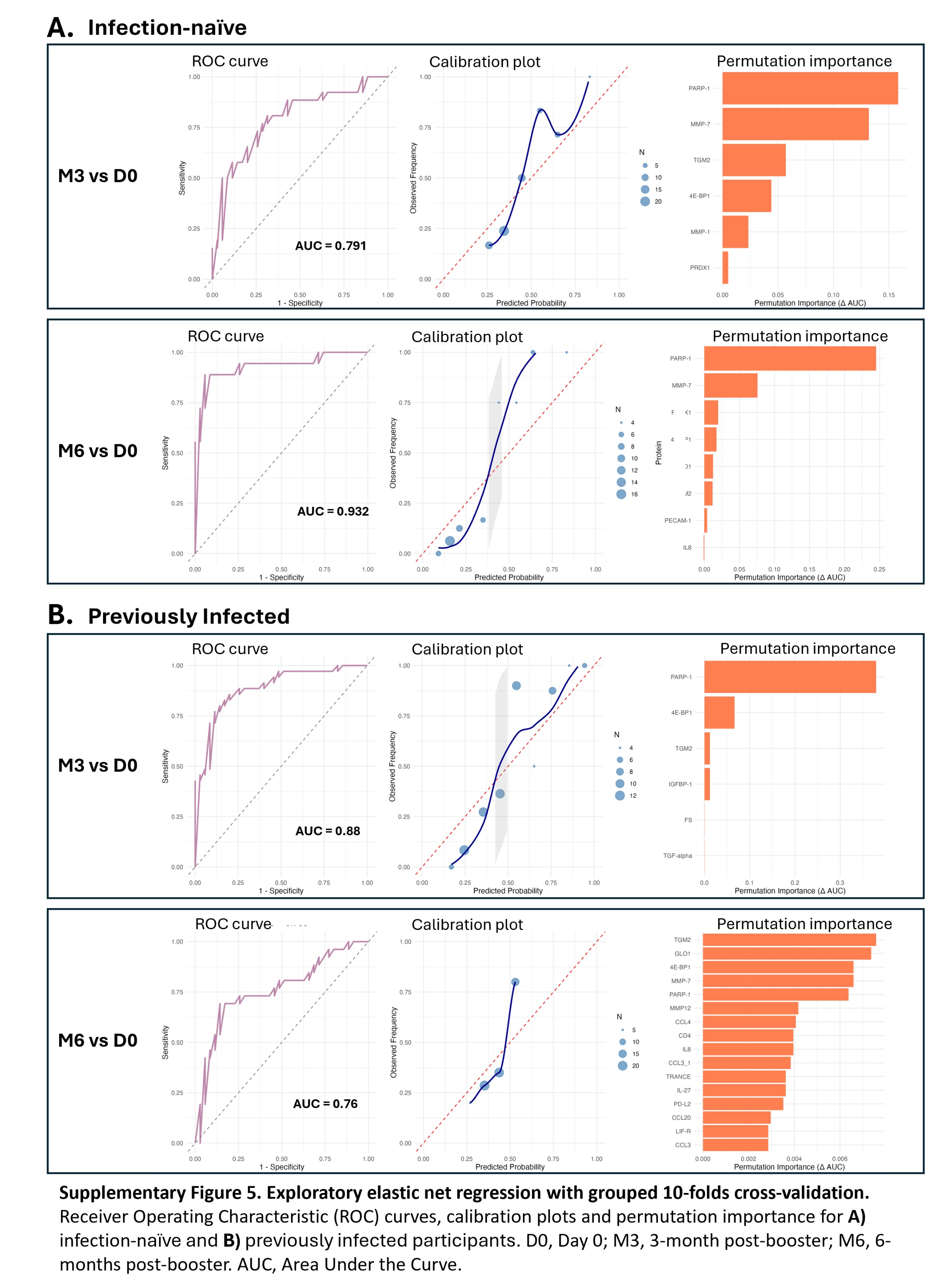

Supplement: Supplementary file 5 [file Image5.jpeg]

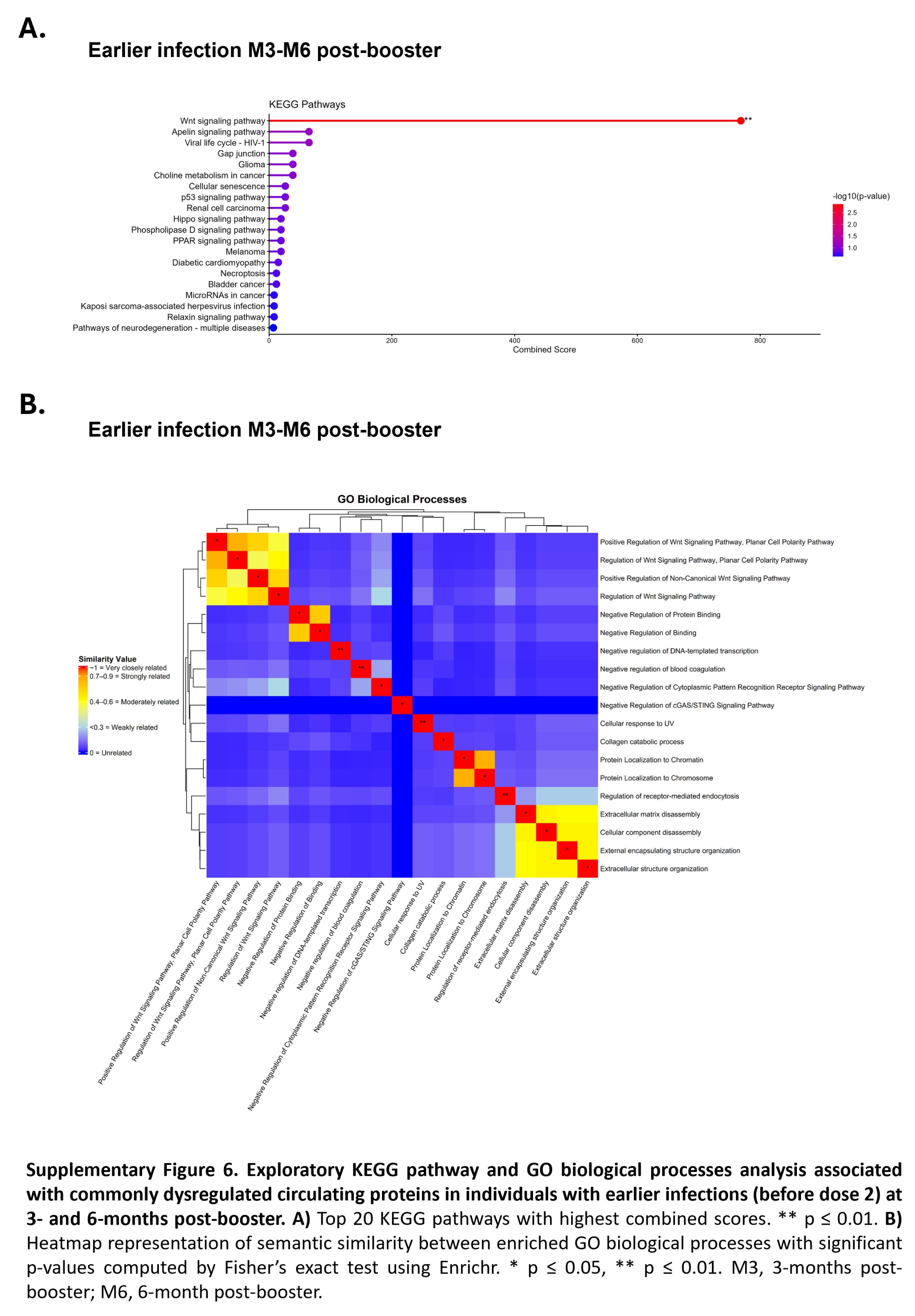

Supplement: Supplementary file 6 [file Image6.jpeg]

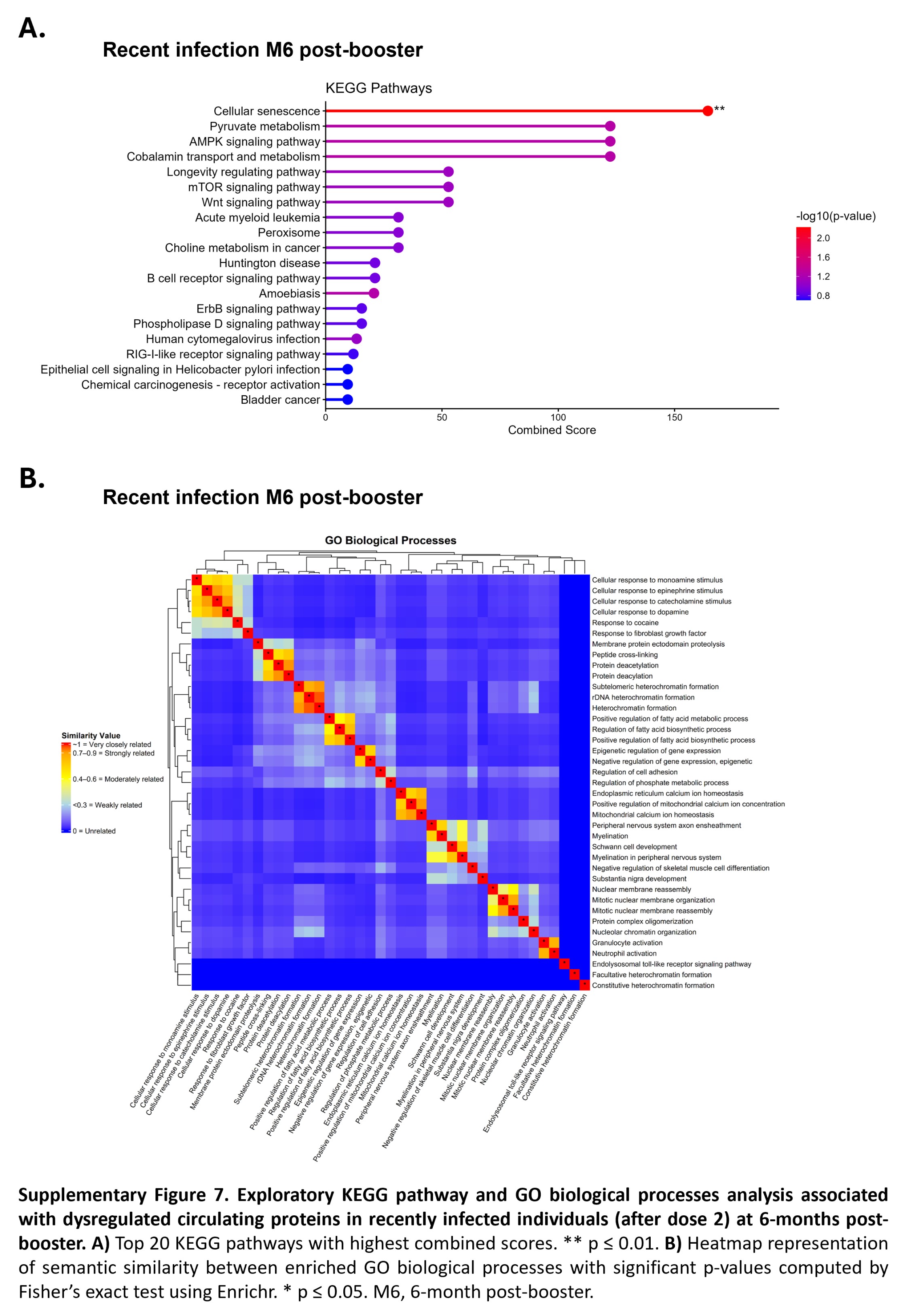

Supplement: Supplementary file 7 [file Image7.jpeg]
